# Multimorbidity patterns and health-related quality of life in Jamaican adults: a cross sectional study exploring potential pathways

**DOI:** 10.3389/fmed.2023.1094280

**Published:** 2023-06-02

**Authors:** Leslie S. Craig, Colette A. Cunningham-Myrie, Katherine P. Theall, Jeanette Gustat, Julie H. Hernandez, David R. Hotchkiss

**Affiliations:** ^1^Department of Medicine, School of Medicine, Tulane University, New Orleans, LA, United States; ^2^Department of Community Health and Psychiatry, University of the West Indies, Mona, Jamaica; ^3^Department of Social, Behavioral, and Population Sciences, School of Public Health and Tropical Medicine, Tulane University, New Orleans, LA, United States; ^4^Department of Epidemiology, School of Public Health and Tropical Medicine, Tulane University, New Orleans, LA, United States; ^5^Department of International Health and Sustainable Development, School of Public Health and Tropical Medicine, Tulane University, New Orleans, LA, United States

**Keywords:** non-communicable diseases (NCD), multimorbidity (MM), health-related quality of life, Jamaica, latent class analysis (LCA)

## Abstract

**Introduction:**

Multimorbidity and health-related quality of life (HRQoL) are intimately linked. Multiple chronic conditions may adversely affect physical and mental functioning, while poorer HRQoL may contribute to the worsening course of diseases. Understanding mechanisms through which specific combinations of diseases affect HRQoL outcomes can facilitate identification of factors which are amenable to intervention. Jamaica, a middle-income country with high multimorbidity prevalence, has a health service delivery system dominated by public sector provision via a broad healthcare network. This study aims to examine whether multimorbidity classes differentially impact physical and mental dimensions of HRQoL in Jamaicans and quantify indirect effects on the multimorbidity–HRQoL relationship that are mediated by health system factors pertaining to financial healthcare access and service use.

**Materials and methods:**

Latent class analysis (LCA) was used to estimate associations between multimorbidity classes and HRQoL outcomes, using latest available data from the nationally representative Jamaica Health and Lifestyle Survey 2007/2008 (*N* = 2,551). Multimorbidity measurement was based on self-reported presence/absence of 11 non-communicable diseases (NCDs). HRQoL was measured using the 12-item short-form (SF-12) Health Survey. Mediation analyses guided by the counterfactual approach explored indirect effects of insurance coverage and service use on the multimorbidity–HRQoL relationship.

**Results:**

LCA revealed four profiles, including a *Relatively Healthy* class (52.7%) characterized by little to no morbidity and three multimorbidity classes characterized by specific patterns of NCDs and labelled *Metabolic* (30.9%), *Vascular-Inflammatory* (12.2%), and *Respiratory* (4.2%). Compared to the *Relatively Healthy* class, *Vascular-Inflammatory* class membership was associated with lower physical functioning (*β* = −5.5; *p* < 0.001); membership in *Vascular-Inflammatory* (*β* = −1.7; *p* < 0.05), and *Respiratory* (*β* = −2.5; *p* < 0.05) classes was associated with lower mental functioning. Significant mediated effects of health service use, on mental functioning, were observed for *Vascular-Inflammatory* (*p* < 0.05) and *Respiratory* (*p* < 0.05) classes.

**Conclusion:**

Specific combinations of diseases differentially impacted HRQoL outcomes in Jamaicans, demonstrating the clinical and epidemiological value of multimorbidity classes for this population, and providing insights that may also be relevant to other settings. To better tailor interventions to support multimorbidity management, additional research is needed to elaborate personal experiences with healthcare and examine how health system factors reinforce or mitigate positive health-seeking behaviours, including timely use of services.

## Introduction

Health-related quality of life (HRQoL), a subjective, multidimensional construct encompassing functioning and well-being in physical, emotional, and social domains (1–3), has been further described by Mayo as a “measure of the value assigned to duration of life as modified by impairments, functional states, perceptions and opportunities, as influenced by disease, injury, treatment and policy” (3, 4). Given chronicity of non-communicable diseases (NCDs), coupled with the potential for poorer physical and mental functioning to erode individual self-management capacity, intensify care demands, and contribute to a worsening of the course of diseases, HRQoL outcomes have important implications for the management of prevalent multimorbidity (i.e., the co-occurrence of two or more NCDs) and prevention of additional conditions (1, 5, 6).

Systematic reviews have consistently documented a strong, inverse relationship between the number of medical conditions and physical HRQoL domains (1, 7). Associations between multimorbidity and mental HRQoL domains are more varied, however, with some studies reporting no statistically significant relationship while others have indicated significant declines in patients with 3 or more concurrent diagnoses (1, 7). Yet, while the body of literature on multimorbidity and HRQoL is growing, commonly cited limitations of available evidence include the frequent exclusion of psychiatric diagnoses from multimorbidity measurement, paucity of data from low- and middle-income country (LMIC) settings, and the reliance on simple counts of diseases (1, 7). Further, despite the existing consensus on the association of prevalent multimorbidity with poorer physical HRQoL domains, there is less agreement regarding the mechanistic pathways underlying poor HRQoL outcomes and the factors that may be amenable to intervention (8).

Aspects of the health system, including instruments that support financial access to care (e.g., insurance coverage) and “hassles” associated with health system interaction (e.g., seeking information, scheduling visits, interacting with health care providers) (8, 9), have been suggested as factors that may present opportunities for intervention along the multimorbidity–HRQoL pathway. Researchers have posited that specific combinations of diseases may have differential effects on patient’s experiences with accessing, using, and enacting, care with potential to differentially affect outcomes (5, 8). A systematic review of qualitative data exploring patient’s subjective experiences of multimorbidity identified financial burdens and frequent healthcare use among the most pressing components of individual experiences in managing the burden and treatment of multimorbidity (9). Specifically, individuals reported that financial pressures were exacerbated by need for private insurance, as this typically shaped user charges and the amount to be reimbursed, while frequent service use negatively impacted the subjective experience of multimorbidity, by serving as a reminder of all health problems currently faced (9).

Improved understanding of the mechanisms through which health system factors, pertaining to financial access and service use, influence quality of life outcomes, can better guide the allocation of resources and organization of care, with important implications for multimorbidity prevention and control. This research used data from the Jamaica Health and Lifestyle Survey 2007/2008 (JHLS-II) to examine the contribution of health system factors to the multimorbidity–HRQoL relationship in Jamaica, a middle-income country in the Caribbean region with a high prevalence of multimorbidity that is disproportionately borne by women (10). Indeed, nationally representative, comprehensive lifestyle surveys such as JHLS-I (2000/2001) and the JHLS-II have been conducted, over the past few years, to better quantify the burden of NCDs and inform targeted action, including the 2012–2017 NCD strategic plan (11). At the time of manuscript preparation, JHLS-III 2016/2017 were not available and the JHLS-II remained the latest available nationally representative dataset for in-depth investigation.

Health service delivery in Jamaica is facilitated through a broad network of primary, secondary, and tertiary care facilities and involves a mix of public and private sectors, with the former being the main provider of public health and hospital services while the latter dominates ambulatory service and pharmaceutical supply provision (10, 12). Recognizing that medication costs account for a substantial portion of out-of-pocket spending, the Government of Jamaica introduced two government programs, funded by government taxes, to enhance financial access to drugs: the Jamaica Drug for the Elderly Programme (JADEP) and the National Health Fund (NHF) (12). JADEP provided subsidies for specific drugs covering select chronic illnesses, for all Jamaican residents over the age of 60, while the NHF provided subsidies to its beneficiaries for the treatment of selected NCDS, without age restrictions (13). Private health insurance schemes are also available and voluntary, with the majority being employer-provided or employer-funded with co-payment by employees.

**Figure 1:**
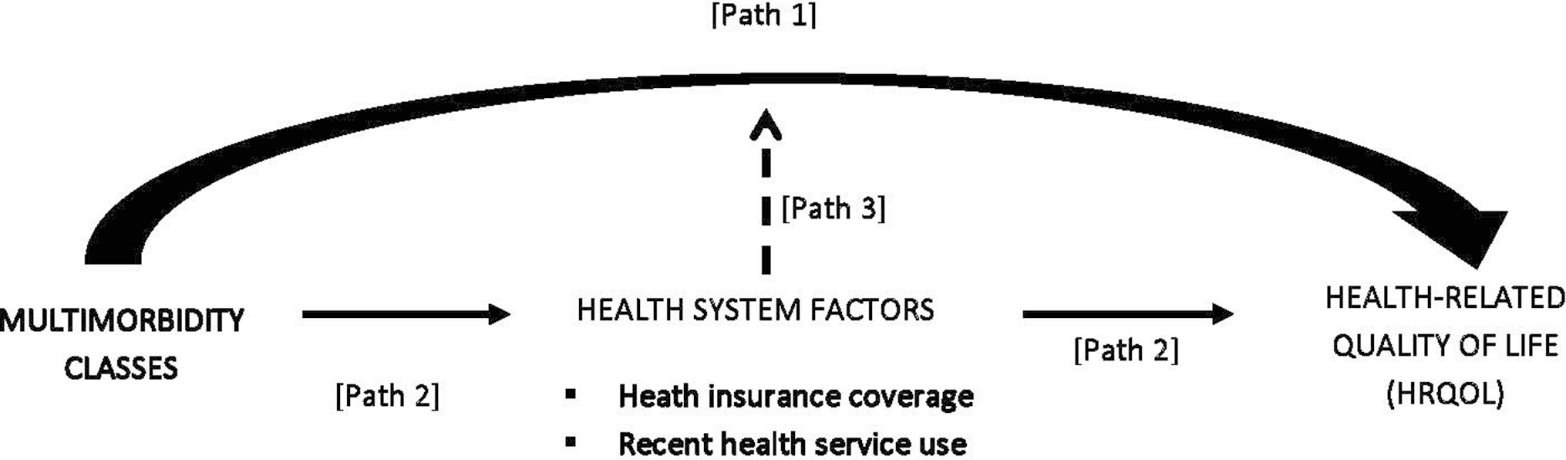
Conceptual framework to guide data analysis (1) Path 1 tests the direct association of multimorbidity class membership on health-related quality of life (HRQoL), (2) Path 2 tests the indirect association of health system factors reflecting financial access to care (i.e., insurance coverage) and health service utilization (i.e., recent health service use) on HRQoL, and (3) Path 3 tests the moderation effect of health system factors on the multimorbidity–HRQoL relationship.

As illustrated in the conceptual framework below (Figure 1), multimorbidity class membership was hypothesized to affect HRQoL (assessed via the SF-12 Health Survey instrument, using summary measures of physical functioning and mental functioning) in the Jamaican population both directly and indirectly, through three main pathways. First, given evidence of a significant inverse relationship between the number of chronic conditions and physical HRQoL domains (1, 7), we hypothesized that multimorbidity classes reflecting a higher number of conditions would be associated with lower physical and mental health functioning (Path 1). Second, given independent effects of the health system reflecting financial access to care (i.e., insurance coverage) and health service utilization (i.e., recent health service use), we hypothesized that by minimizing financial stress and enabling better health-seeking behaviour, insurance coverage would mediate (i.e., lessen) the impact of multimorbidity on physical and mental dimensions of HRQoL (Path 2) while, due to challenges navigating the health system, recent service use would mediate (i.e., intensify) the multimorbidity–HRQoL relationship via a negative effect on mental health functioning (Path 2). Finally, we hypothesized that interactions between multimorbidity and health system factors would moderate the multimorbidity–HRQoL relationship, either by mitigating (in the case of insurance coverage) or reinforcing (in the case of health service use) its effects on health outcomes (Path 3).

## Materials and methods

### Data

Secondary analysis of JHLS-II data was performed. Study design, recruitment, sampling, and data collection procedures for the JHLS-II have been previously described (13). Briefly, 2,848 individuals, 15–74 years old of age, were recruited between November 2007 and March 2008 to participate in this nationally representative, interviewer-administered, cross-sectional survey (13, 14). Participant recruitment was guided by a multi-stage cluster sampling design and based on random selection of clusters (enumeration districts) proportional to the size of the population within all parishes in Jamaica (13). Trained interviewers used structured questionnaires and standardized protocols to collect demographic, medical history, and health behaviour data, and obtain physical (i.e., height, body weight, hip circumference, and waist circumference), and biological (i.e., blood pressure, blood glucose, and total cholesterol) measurements (13, 14).

### Measures

In this study, four categories of variables were operationalized:

1. **Exposure**: multimorbidity class membership,
2. **Outcomes**: HRQoL, relating to physical and mental domains,
3. **Mediators of the multimorbidity–HRQoL relationship**: health system factors reflecting financial access to care and health care utilization, and
4. **Covariates**: covariates included socio-demographic characteristics, economic circumstances, and health behaviours with well-documented associations with quality-of-life outcomes.

#### Multimorbidity class membership

Binary indicators reflecting the presence/absence of 11 NCDs were used to categorize the population into latent class (i.e., multimorbidity) segments, based on the relative probabilities that individuals within the class have a particular NCD. These indicators of multimorbidity class membership have been previously described (15, 16). In brief, only those NCDs with the greatest burden in this population, defined as a prevalence of 1% or higher in each sex, were included as indicators: hypertension, obesity, hypercholesterolemia, diabetes, asthma, arthritis, cardiovascular disease, mental health disorders, chronic obstructive pulmonary disease (COPD), stroke, and glaucoma. Self-reported diagnosis of bronchitis/pneumonia served as a proxy for COPD and, in keeping with guidance on multimorbidity measurement (17), cardiovascular (i.e., heart disease, myocardial infarction, and circulation problems) and mental health (i.e., depression, anxiety, psychosis, and other mental health problems) conditions were grouped together to reflect cardiovascular diseases and mental health disorders, respectively.

#### Health-related quality of life (HRQoL)

HRQoL was measured using the SF-12 Health Survey instrument, a 12-item short-form that captures eight dimensions of health (i.e., Physical Functioning, Role Physical, Bodily Pain, General Health, Vitality, Social Functioning, Role Emotional, and Mental Health) and can be used to generate two summary subscale scores reflecting physical and mental HRQoL functioning: the Physical Component Summary (PCS-12) and Mental Component Summary (MCS-12) scores (18). The SF-12 captures eight dimensions of health, with the PCS-12 generally measuring Physical Functioning, Role Limitations due to Physical Health Problems, Bodily Pain, and General Health domains, while the MCS-12 predominantly reflects Vitality (i.e., energy/fatigue), Social Functioning, Role Limitations due to Emotional Problems, and Mental Health (i.e., psychological distress and psychological well-being) (19). Summary HRQoL measures of physical and mental health remain a valuable approach to the measurement of health and functional status, and recommendations urge use of traditional SF-12 scoring and procedures for their derivations (20). In accordance with these recommendations (19), PCS-12 and MCS-12 subscale scores were weighted, aggregated and standardized, to achieve a mean score of 50 and standard deviation (SD) of 10, and allow for meaningful comparison with each other (19). PCS-12 and MCS-12 scores were set to missing if an individual was missing data on any one of the SF-12 items, and mean substitution for missing values used. Higher scores were indicative of better HRQoL (2).

#### Health system factors

Individual-level health system factors included indicators of health insurance coverage and health service use. For health insurance coverage, binary indicators of public coverage (based on enrolment in either the NHF or JADEP) and private insurance coverage reflected better financial access to needed medications and health services, respectively. Based on data availability, a binary indicator of recent health service use was created using a proxy measure based on reports of the last blood pressure measurement being taken in the 6 month period prior to the survey.

#### Covariates

Socio-demographic, economic, and behavioural factors noted to impact quality of life outcomes were defined (1). Self-reported socio-demographic characteristics included age in years (treated as continuous), sex (male/female), full-time employment (yes/no), and having attained at least secondary level education or higher (yes/no). For economic circumstances, principal component analysis (PCA) was used to generate quintiles ranging from poorest to wealthiest, based on (yes/no) responses to questions on ownership of household assets (i.e., gas/electric stove, refrigerator or freezer, microwave oven, telephone, radio, television set, cable, satellite dish, bicycle, motorbike, car, computer, washing machine, sewing machine, fan, air conditioner, compact disk (CD) player, stereo equipment, record player, and video cassette recorder), and living conditions (i.e., number of members per sleeping room). This wealth index was then dichotomized to reflect discrete high vs. low groups, specifying those in the top 60% versus the bottom 40% (21). Health behaviours reflected past or current tobacco use (yes/no), current alcohol drinkers (yes/no) and low physical activity levels (yes/no). Physical activity levels were defined using metabolic equivalent (MET) levels based on walking, moderate-intensity activity, and vigorous-intensity activity scores from the International Physical Activity Questionnaire (IPAQ)-Short Form (22). Based on established cut-offs, categories reflecting high (i.e., ≥7 or more days of any combination of walking, moderate-or vigorous-intensity activities accumulating at least 3,000 MET-minutes/week), moderate (i.e., ≥5 days of any combination of walking, moderate-or vigorous-intensity activities achieving a minimum of at least 600 MET-minutes/week) or low (i.e., no activity or some activity reported but not enough to meet moderate or high levels) levels of activity were derived (22), and the former two categories collapsed to create the binary, low physical activity levels indicator.

### Ethics approval and consent to participate

The JHLS-II survey and study protocol were approved by the Ministry of Health, Jamaica and the University of the West Indies/University Hospital of the West Indies Ethics Research Committee (ECP 169, 14/15). Written informed consent was obtained from all adult study participants and, for participants under 18 years of age, written consent was received from a parent and/or legal guardian. All study procedures were performed in accordance with institutional guidelines and confidentiality of all participants and national data were protected within legal limits.

### Statistical approach

Descriptive statistics were examined using proportions for categorial variables and means with 95% confidence intervals (CIs) for continuous variables. Base sampling weights, calculated as the product of the inverse of the probability of selecting a household and the inverse of the probability of selecting a primary sampling unit, were applied to account for sampling design and non-response. Further, post-stratification weights, calculated as the number of persons in the Jamaican population between the ages of 15–74 years, represented by each individual in the sample within 5 year age-sex categories, were used. In keeping with recommendations (23), regressions were unweighted. All analyses were performed using Stata v.15 software (StataCorp, College Station, TX).

#### Latent class analysis (LCA)

Multimorbidity classes were identified using LCA, a reductionist strategy that uses a person-centred approach to identify segments of the population with diverging disease profiles (24). First, a series of models, with a progressively increasing number of latent classes, were fit to the data. Baseline model selection was driven by comparison of model fit statistics (i.e., likelihood-ratio G^2^ statistic and parametric bootstrap likelihood ratio test) and information criteria (i.e., Akaike Information Criteria (AIC), Bayesian Information Criteria (BIC), adjusted BIC) in addition to visual inspection of probability plots to evaluate the meaningfulness and distinctiveness of resultant latent class solutions (25). A description of the methodology used to determine baseline model selection can be found in Craig et al. 2020 (15).

Next, a Bayes’ theorem-based approach was used to estimate the effect of multimorbidity class membership on physical and mental HRQoL domains. This involved fitting the latent class model with the outcome included as a covariate, and then using Bayes’ theorem to reverse the direction of the effect and empirically derive the class-specific distribution of mean PCS-12 and MCS-12 scores, via kernel density estimation (26). Pairwise tests and corresponding *values of p*, with statistical significance set at *p* < 0.05, were used to compare each multimorbidity pattern with the reference class and test the null hypothesis that scores were equal. All statistical analyses were carried out via Stata v.15 software, using the LCA Stata Plugin (27) and the LCA_Distal_BCH Stata function (28) as needed.

#### Multivariable regression analysis

Following LCA, individuals were assigned to their best fit class based on their maximum posterior probability. Multivariable regression methods were then used to further examine the multimorbidity–HRQoL relationship, separately for physical and mental domains, while controlling for important confounders known to affect quality of life outcomes (7).

#### Mediation analyses

To investigate the role of health system factors on HRQoL outcomes, the counterfactual approach to mediation analysis, developed by Valeri and VanderWeele (29), was used. This approach allows the total effect of multimorbidity on HRQoL to be decomposed into direct and indirect effects, using models with interactions (e.g., between multimorbidity classes and health system factors) and non-linearities (e.g., binary mediators such as insurance coverage and recent health service use) (29). Analyses were performed in Stata using the *paramed* program, which is based on the mediation macro developed by Valeri and VanderWeele (29). Multimorbidity classes were recoded as separate binary indicators (i.e., *Metabolic*, *Vascular-Inflammatory*, and *Respiratory*) to accommodate programming requirements for operationalization of the exposure variable. To satisfy identifiability assumptions regarding no confounding (29), key socio-demographic, economic, and health behaviour covariates were included in the regression. Potential interactions between multimorbidity classes and health system factors were tested for their effects on HRQoL and included where significant. Natural direct and indirect effects were estimated by fitting a linear regression model for the continuous outcome and a logistic regression model for the binary mediator, respectively (29). From these combined models, estimates for the natural direct effects, natural indirect effects, and total effects (i.e., the sum of the natural direct and indirect effects) were determined, along with bias-corrected bootstrap confidence intervals (via bootstrap procedures with 1,000 replications) (29). The proportion of the multimorbidity–HRQoL relationship mediated through health system factors was calculated as the ratio of the natural indirect effect to the total effect.

## Results

### Sample characteristics

#### Multimorbidity latent classes

Only participants with complete information on the presence or absence of the 11 NCD multimorbidity indicators were included in the analytic sample. There were no statistically significant differences between those with complete (*n* = 2,551) and those with missing data (*n* = 311) with respect to age, sex, or region of residence. Fit statistics, information criteria, entropy scores and model interpretability together suggested that the four-class solution was the optimum baseline model. Briefly, the G^2^ statistic, Akaike Information Criteria (AIC) and the adjusted Bayesian Information Criteria (BIC) reached their lowest in the four-class model. The four-class solution’s entropy score also indicated precision in class prediction and, upon examination, allowed for meaningful interpretation of resultant latent classes. Latent class prevalences and item-response probabilities (i.e., the estimated probability of reporting a particular NCD, given membership in a particular latent class) for the four-class model have been fully described in an earlier publication (15) and are illustrated in Table 1. In summary, the final classes were labelled: *Relatively Healthy* (52.7%) – characterized by individuals with very low probability of any of the 11 NCDs; *Metabolic* (30.9%) – characterized by individuals with a high probability of hypertension and obesity, and somewhat moderate probability of hypercholesterolemia; *Vascular-Inflammatory* (12.2%) – characterized by individuals with the highest probability of hypertension, obesity, hypercholesteremia and diabetes, in addition to a high probability of arthritis and cardiovascular disease; and *Respiratory* (4.2%) – characterized by individuals with the highest probability of asthma and COPD.

**Table 1:** Four-latent-class model of multimorbidity.

	Latent class			
	1	2	3	4
	Relatively healthy	Metabolic	Vascular inflammatory	Respiratory
*Latent class prevalences*	0.53	0.31	0.12	0.04
*Item-response probabilities*	**Probability of a Yes response**			
Hypertension	0.05	**0.58**	**0.80**	0.14
Obesity	0.20	**0.39**	**0.56**	**0.53**
Hypercholesterolemia	0.05	0.23	**0.36**	0.20
Diabetes mellitus	0.00	0.17	**0.36**	0.08
Asthma	0.07	0.02	0.06	**0.45**
Arthritis	0.03	0.04	**0.43**	0.05
Cardiovascular disease	0.02	0.00	**0.41**	0.10
Mental health disorders	0.02	0.01	0.07	0.12
COPD	0.02	0.00	0.05	**0.41**
Stroke	0.00	0.01	0.10	0.00
Glaucoma	0.00	0.02	0.07	0.00
Mean number of NCDs reported (95% CI)	0.40 (0.36, 0.45)	1.60 (1.53, 1.67)	3.40 (3.23, 3.56)	2.86 (2.72, 3.00)

*Item-response probabilities ≥ 0.35 in boldface to facilitate interpretation.

#### Health system factors

**Figure 2:**
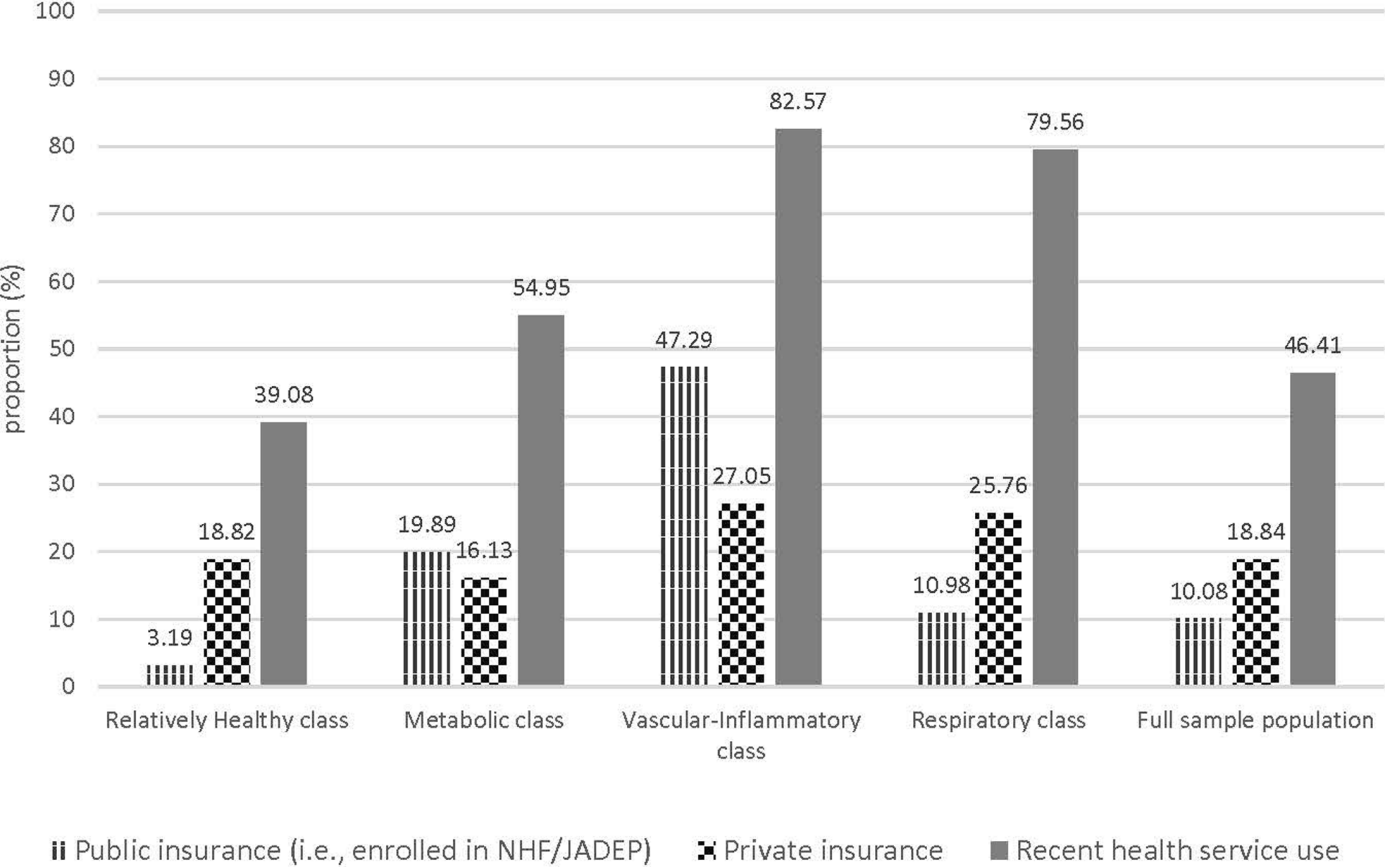
Health Insurance coverage and recent health service encounters, according to class membership.

A significantly larger proportion of the sample had private health insurance compared to public coverage through subsidies (18.8% vs. 10.1%; *p* = 0.008) (Figure 2). Specifically, 13.7% of the study population had enrolled in the NHF while only 4.2% had enrolled in JADEP; among those over 60 years, 27.0% were enrolled in JADEP. Compared to their *Relatively Healthy* counterparts (18.8%), the *Metabolic* class was less likely (*p* = 0.034) and the *Vascular-Inflammatory* class more likely (*p* = 0.003) to own private insurance. All multimorbidity classes were significantly more likely than their *Relatively Healthy* counterparts (*p* ≤ 0.001) to have public insurance coverage (i.e., be enrolled in either of the government subsidized programs). Similarly, all multimorbidity classes were significantly more likely than their *Relatively Healthy* counterparts to have had a recent health service visit (*p* < 0.001).

#### Distribution of mean HRQoL subscale scores

Mean PCS-12 and MCS-12 scores for the survey population were 51.3 (SD = 8.5) and 49.5 (SD = 9.0), respectively. Table 2 presents mean PCS-12 and mean MCS-12 scores, given latent class membership. Individuals in the *Metabolic* and *Vascular-Inflammatory* classes scored 2.7 (*p* < 0.001) and 13.4 (*p* < 0.001) points lower on the PCS-12 subscale, respectively, than their *Relatively Healthy* counterparts. There were no statistically significant differences in mental health functioning scores according to latent class membership.

**Table 2:** Mean HRQoL subscale scores, according to latent class.

Latent class	Physical component summary (PCS-12) score			Mental component summary (MCS-12) score		
	Mean score	95% CI	value of *p*	Mean score	95% CI	value of *p*
Relatively healthy	53.85	(53.37, 54.33)	[ref]	49.56	(48.94, 50.19)	[ref]
Metabolic	51.13	(50.13, 52.12)	<0.001	50.49	(49.50, 51.48)	0.179
Vascular-inflammatory	40.48	(38.64, 42.32)	<0.001	48.56	(47.07, 50.05)	0.211
Respiratory	52.56	(49.46, 55.65)	0.435	45.62	(41.49, 49.75)	0.074

*p*-values based on Wald chi-squared tests.

### Multivariate regression analyses

Following assignment of individuals to their best fit class based on their maximum posterior probability, regression estimates indicated that, compared to those in the *Relatively Healthy* class, membership in the *Vascular-Inflammatory* class (*β* = −5.5; *p* < 0.001) was associated with significantly lower PCS-12 scores (Table 3). For MCS-12, individuals in the both *Vascular-Inflammatory* class (*β* = −1.7; *p* < 0.05) and *Respiratory* class (*β* = −2.5; *p* < 0.05) had significantly lower mental functioning scores than their *Relatively Healthy* counterparts.

**Table 3:** Multivariate regression analysis of effect of multimorbidity class membership on HRQoL outcomes.

	Physical component summary (PCS-12) score (*N* = 2,499)	Mental component summary (MCS-12) score (*N* = 2,499)
	*β* (95% CI)	*β* (95% CI)
Multimorbidity class		
Relatively healthy	[ref]	[ref]
Metabolic	−0.13 (−0.89, 0.62)	−0.27 (−1.16, 0.63)
Vascular-inflammatory	−5.48*** (−6.66, −4.29)	−1.69* (−3.09, −0.28)
Respiratory	−0.35 (−2.41, 1.70)	−2.53* (−4.97, −0.09)
Age, years	−0.11*** (−0.14, −0.09)	0.06*** (0.03, 0.09)
Female sex	−0.26 (−0.99, 0.48)	−2.60*** (−3.47, −1.73)
Secondary level or higher	1.39*** (0.64, 2.15)	−0.67 (−1.57, 0.23)
Employed full-time	1.46*** (0.84, 2.07)	0.21 (−0.53, 0.94)
Top 60% wealth quintile	0.51 (−0.13, 1.16)	1.42*** (0.66, 2.19)
Currently use alcohol	0.66* (0.00, 1.32)	−0.76 (−1.55, 0.02)
Past or present smoker	−0.39 (−1.10, 0.33)	−1.88*** (−2.73, −1.03)
Low levels of physical activity	−0.93** (−1.53, −0.32)	−0.55 (−1.27, 0.17)
Private insurance coverage	−0.18 (−1.00, 0.65)	1.11* (0.14, 2.09)
Public insurance coverage	−2.69*** (−3.66, −1.73)	0.17 (−0.97, 1.31)
Recent health service use	−0.53 (−1.18, 0.11)	−1.03** (−1.80, −0.27)

CI, confidence interval. Estimates based on non-missing data (<1% missing across all variables). *p < 0.05, **p < 0.01, ***p < 0.001.

Results of the multivariate model further indicated that higher educational attainment (*β* = 1.4; *p* < 0.001) and full-time employment (*β* = 1.5; *p* < 0.001) were associated with higher PCS-12 scores, while low levels of physical activity (*β* = −0.9; *p* < 0.01) were associated with lower PCS-12 scores. Being in the top 60% wealth quintile (*β* = 1.2; *p* < 0.001) was associated with higher MCS-12 scores. Conversely, being female (*β* = −2.6; *p* < 0.001) and self-reporting past or current use of tobacco (*β* = −1.9; *p* < 0.001) were associated with lower MCS-12 scores. Advancing age was associated with lower PCS-12 scores (*β* = −0.1; *p* < 0.001), but higher MCS-12 scores (*β* = 0.1; *p* < 0.001). Public insurance coverage through subsidies was associated with lower PCS-12 scores (*β* = −2.7; *p* < 0.001) while private insurance coverage was associated with higher MCS-12 scores (*β* = 1.1; *p* < 0.05). Recent health service use was associated with lower scores on the MCS-12 subscale (*β* = −1.0; *p* < 0.01).

### Mediation analyses

Results of the mediation analyses for PCS-12 and MCS-12 scores are presented in Tables 4, 5, respectively. As noted above, significant total effects were observed between the *Vascular-Inflammatory* class and PCS-12 scores, while lower MCS-12 scores were significantly associated with membership in both the *Vascular-Inflammatory* and *Respiratory* classes (Table 3).

**Table 4:** Mediation analysis: effects of multimorbidity on the PCS-12 subscale scores.

	Natural direct effect	Natural indirect effect	Marginal total effect
	*β* (95% CI^ϯ^)	*β* (95% CI^ϯ^)	*β* (95% CI^ϯ^)
*Vascular-inflammatory class*			
Mediator: recent service use^a^^,¥^	−5.38*** (−6.80, −3.98)	−0.17 (−0.26, 0.02)	−5.49*** (−6.93, −4.15)
Mediator: private insurance^b^	−5.66*** (−7.17, −4.24)	0.16 (−0.05, 0.57)	−5.50*** (−6.93, −4.08)
Mediator: public insurance^c^^,¥^	−5.38*** (−6.73, −3.86)	−0.27** (−0.53, −0.14)	−5.66*** (−7.03, −4.17)

PCS-12, physical component summary score. ^ϯ^Bias-corrected confidence interval. ^¥^The exposure–mediator interaction was not significant at the *p* < 0.05 level and not included in the model.

a Adjusted for age, sex, education, employment, wealth, current alcohol use, past or present smoking status, physical activity level, public insurance coverage, and private insurance coverage.

b Adjusted for age, sex, education, employment, wealth, current alcohol use, past or present smoking status, physical activity level, public insurance coverage, and recent service use.

c Adjusted for age, sex, education, employment, wealth, current alcohol use, past or present smoking status, physical activity level, private insurance coverage, and recent service use.

**Table 5:** Mediation analysis: effects of multimorbidity on the MCS-12 subscale scores.

	Natural direct effect	Natural indirect effect	Marginal total effect
	*β* (95% CI^ϯ^)	*β* (95% CI^ϯ^)	*β* (95% CI^ϯ^)
*Vascular-Inflammatory class*			
Mediator: recent service use^a^^,¥^	−1.44* (−2.85, −0.11)	−0.22* (−0.42, −0.08)	−1.66* (−3.01, −0.37)
Mediator: private insurance^b^^,¥^	−1.44* (−3.00, −0.19)	0.09 (0.02, 0.25)	−1.35* (−2.90, −0.09)
Mediator: public insurance^c^^,¥^	−1.44* (−2.82, −0.11)	0.01 (−0.10, 0.15)	−1.42* (−2.75, −0.13)
*Respiratory class*			
Mediator: recent service use^a^^,¥^	−2.24 (−5.48, 0.55)	−0.25* (−5.39, −0.06)	−2.49* (−5.57, 0.33)
Mediator: private insurance^b^^,¥^	−2.24 (−5.44, 0.51)	−0.02 (−0.12, 0.06)	−2.25 (−5.48, 0.43)
Mediator: public insurance^c^	−2.10 (−6.24, 1.48)	−0.38 (−2.65, 1.45)	−2.48 (−5.52, 0.63)

MCS-12, mental component summary score. ^ϯ^Bias-corrected confidence interval. ^¥^Exposure–mediator interactions were not significant at the *p* < 0.05 level and not included in the models.

a Adjusted for age, sex, education, employment, wealth, current alcohol use, past or present smoking status, physical activity level, public insurance coverage, and private insurance coverage.

b Adjusted for age, sex, education, employment, wealth, current alcohol use, past or present smoking status, physical activity level, public insurance coverage, and recent service use.

c Adjusted for age, sex, education, employment, wealth, current alcohol use, past or present smoking status, physical activity level, private insurance coverage, and recent service use.

For physical functioning, there was some mediation by public insurance coverage (Table 4), with 4.8% of the total effect of the *Vascular-Inflammatory* class on PCS-12 scores mediated by enrolment in government subsidized programs (*β*_total_ = −5.7, *p* < 0.001; *β*_indirect_ = −0.3, *p* < 0.01). There were no statistically significant indirect effects mediated by recent service use or private insurance coverage.

With regard to mental health outcomes, the total effects of the *Vascular-Inflammatory* class and the *Respiratory* class on MCS-12 scores both appeared to be mediated by recent health service use (Table 5). The proportions mediated through recent service use were 13.3 and 10.0%, for the association between MCS-12 scores and membership in the *Vascular-Inflammatory* class (*β*_total_ = −1.7, *p* < 0.05; *β*_indirect_ = −0.2; *p* < 0.05) and the *Respiratory* class (*β*_total_ = −2.5, *p* < 0.05; *β*_indirect_ = −0.3; *p* < 0.05), respectively. There were no statistically significant indirect effects on MCS-12 scores by insurance coverage for either the *Vascular-Inflammatory* or *Respiratory* classes.

## Discussion

This study used LCA to explore the distribution of HRQoL outcomes conditional on multimorbidity class membership and explore the role of health system factors in mediating this relationship. Findings indicate that beyond the accumulation of an increasing *number* of conditions, risk of adverse HRQoL outcomes is further modified by the *types* of disease combinations. Specifically, the study illustrated that specific disease profiles are differentially associated with poorer physical functioning, with the *Metabolic* class and the *Vascular-Inflammatory* class scoring 2.7 (*p* < 0.001) and 13.4 (*p* < 0.001) points lower, respectively, than their counterparts in the *Relatively Healthy* class. After controlling for important confounders, results continued to implicate multimorbidity patterns as important determinants of physical functioning and well-being, with membership in the *Vascular-Inflammatory* class associated with lower PCS-12 scores, compared to membership in the *Relatively Healthy* class. For mental health functioning, results of the multivariate regression analyses indicated that multimorbidity patterns were also important determinants of psychological well-being, with significant inverse relationships observed between MCS-12 scores and membership in both the *Vascular-Inflammatory* and *Respiratory* classes. Given that the mean number of NCDs reported among members of the *Vascular-Inflammatory* and *Respiratory* classes was 3.40 and 2.86, respectively, this finding is further in keeping with evidence of poorer functioning, along mental HRQoL domains, following 3 or more concurrent diagnoses (1). Findings also indicated low levels of private and public insurance coverage and alluded to relatively high health system interactions (i.e., high proportions of respondents with a recent health care visit), particularly among those with varying patterns of multimorbidity.

Findings from this study indicate that membership in the *Vascular-Inflammatory* class (characterized by very high probability of hypertension, obesity, hypercholesteremia, and diabetes, in addition to an increased likelihood of self-reported arthritis and cardiovascular disease) is associated with particularly increased vulnerability to poor quality of life outcomes, across both physical and mental domains. A Canadian study among 238 primary care patients (18 years or older) explored differential impacts of varying combinations of conditions, grouped by anatomical domain, with HRQoL outcomes and similarly concluded that vascular, upper gastro-intestinal and musculoskeletal systems have strong negative effects on physical dimensions of HRQoL (30). Given that the PCS-12 subscale is heavily weighted towards HRQoL aspects reflecting physical functioning, general health and pain, these findings suggest that interventions targeting physical therapy and pain management may promote better disease control and quality of life outcomes for members of the *Vascular-Inflammatory* class. Importantly, researchers have noted that living with a chronic disease does not preclude mental well-being attainment and urge identification of individual strengths, assets and motivations to inform resource-centered interventions (31). Accordingly, given that the MCS-12 subscale is heavily weighted towards HRQoL aspects reflecting energy, social engagement, and emotional well-being, more investigation is needed to understand how *Vascular-Inflammatory* and *Respiratory* patterns differentially affect energy levels, social functioning, and mental well-being, in order to support more holistic care interventions that promote better psychosocial adaption and resilience. A systematic review of 10 randomized controlled trials of interventions to improve outcomes for patients with multimorbidity, indicated that strategies targeting quality of life outcomes and functional difficulties were most promising, with evidence of a statistically significant reduction in mortality 2 years post-intervention (32). Given that individuals in the *Vascular-Inflammatory* class appear to be at greatest risk for both poor physical and mental health functioning, further investigation into the role of health system factors in reinforcing or mitigating poor outcomes is also warranted.

In considering entry points for interventions to support improved multimorbidity management via better physical functioning and emotional wellbeing, results of mediation analyses highlight possible pathways. There was a significant mediated effect through recent health service use, on the relationship between multimorbidity and mental health functioning, for individuals in both the *Vascular-Inflammatory* and *Respiratory* classes. This finding corroborated the hypothesis that prior health care use would mediate the multimorbidity–HRQoL association negatively, reflecting personal frustrations, and health system hassles that have been reported in the international literature (9) among primary care patients with prevalent multimorbidity. A small, yet statistically significant mediated effect through public insurance coverage was also observed, indicating that enrolment in either the NHF or JADEP was associated with lower physical functioning. This finding may reflect a limitation of cross-sectional analyses which challenge temporal ordering of exposure and outcome, as it is not clear why access to medications would be associated with lower physical functioning. One possible explanation lies in the nature of these government subsidized programs, where the JADEP caters to populations over the age of 60 years (where physical limitations are more common) while the NHF is a needs-based program with enrolment conditional on the confirmed diagnosis of disease. It is thus likely that persons seek the benefits of these programs after their illnesses have reached a more advanced stage where physical limitations become apparent and pharmaceutical intervention is more urgent. Findings may also reflect sub-optimal utilization of the NHF or JADEP programs, as study data suggest relatively low enrolment, hinting at minimal uptake of benefits provided by these government schemes. Regardless, this finding underscores the need for timely access to services and medications to better support the management of multiple co-existing morbidities and prevent the accumulation of new ones.

Researchers have suggested that multiple chronic conditions may act additively or synergistically to adversely affect health outcomes (1, 30, 33). Data from middle-income countries indicate that multimorbidity (based on disease counts) is positively associated with higher levels of healthcare utilisation (34) but inversely associated with quality-of-life outcomes (35). Further, chronicity of NCDs and the dynamic nature of health and well-being predispose multimorbid individuals to negative feedback cycles, where poorer quality of life outcomes reduce capacity to manage multiple conditions and facilitate the accumulation of new ones (5). Comprehensive care frameworks acknowledge HRQoL as a critical patient-related outcome, with implications for reducing the burden of prevalent conditions and promoting treatment compliance (36). Minimizing risk of adverse health outcomes in this vulnerable sub-group of persons with multimorbidity requires a deeper look into health-seeking behaviours, coping strategies and the role of enabling factors (e.g., insurance) in alleviating concerns regarding access to and use of care. While national efforts to reduce access barriers (i.e., following establishment of the NHF and JADEP programs) were implemented in 2008, when the Government of Jamaica abolished user fees in public facilities, the ensuing increased demand has challenged service quality, with reports of long wait times, insufficient supplies and inadequate human resources, prompting private care-seeking among Jamaicans of all income groups, including the poorest quintiles (12). In the absence of systematic research on the role of health system factors on multimorbidity management and HRQoL outcomes, anecdotal reports suggest that public health clinics in Jamaica are typically overburdened with serving large numbers of the population, such that clients experience difficulty scheduling appointments outside of a usual 3–6 month window in addition to challenges concerning inconsistent drug availability and limited surgical intervention within the public domain. Future qualitative work would allow for better elaboration of the personal experiences of Jamaicans with prevalent multimorbidity, as well as challenges and concerns regarding financial access to services and medication.

### Strengths and limitations

There are several strengths and limitations of this study. At the time of writing, these data represented the latest available population-level collection, since results of the JHLS-III were not yet available, and the technical report not finalized. Given that NCDs continue to dominate the health and economic burden for Jamaica (37), and the wider Caribbean region (38, 39), findings from this study remain relevant by providing insights for public health planning, policy, and clinical intervention. The cross-sectional study design challenges the temporal ordering assumptions needed for mediation analysis, which require that the exposure (i.e., multimorbidity) preceded the mediator (i.e., health system factors) which, in turn, preceded the outcome (i.e., scores on the HRQoL subscales). The Bayes’ theorem-based approach is recommended as a robust technique for modelling the effect of latent classes on a distal outcome (26), and use of this approach to examine the distribution of mean PCS-12 and MCS-12 scores, conditional on class membership, is a strength of this study. Yet, this model does not allow for statistical control of potential confounders or formal tests of mediation. Further, while the maximum probability assignment method has been widely used throughout the literature to estimate the associations between latent class membership and distal outcomes and has been noted to produce less biased estimates compared to other classify-analyze approaches, this method fails to account for uncertainty in class membership (26). Simulation studies indicate that maximum probability assignment approaches often attenuate effects of the exposure on the outcome (26), suggesting that the impact of multimorbidity patterns on HRQoL outcomes may be even greater than estimated here. These limitations must be borne in mind in interpretation of the results.

Limitations in the definition and measurement of indicators must also be considered. Ideally, health service use would have been operationalized using a measure reflecting the number of visits made within a specified period, to better capture frequency of use and the increased service utilization that has come to be associated with multimorbidity. The recent health care service use indicator used in this study was a convenience measure (based on a blood pressure measurement by a health professional in the past 6 months), with selection determined by JHLS-II questionnaire availability. Limitations of this binary indicator are acknowledged, including failure to capture the type of health care service used (e.g., primary, secondary, and tertiary), the purpose of the visit (i.e., routine vs. emergency consultation) or the number of health visits made within a given time period. In addition, few indicators were dichotomized based on arbitrary cut-points (e.g., top 60% vs. bottom 40% wealth quintile). Further, despite inclusion of several covariates deemed relevant, residual confounding due to omission of potentially important social (e.g., social support), disease-related (e.g., severity of diseases, number of medications being used), and health system (e.g., quality of care, hospitalization) factors, beyond those included in the study, may have occurred. Studies note that important factors impacting the multimorbidity–HRQoL relationship may include the presence of coexisting acute conditions, time since diagnosis of chronic diseases, and the prognosis of health conditions (1). These variables were not available in the dataset and not accounting for them could have potentially biased the effect estimates observed here.

## Conclusion

This study identified differential effects of specific combinations of diseases on HRQoL outcomes, demonstrating the clinical utility and epidemiological value of latent classes (i.e., multimorbidity patterns) in estimating risk of poor mental and physical functioning, and highlighting health system factors that represent opportunities for intervention. In order to better tailor interventions to support management of multiple conditions, additional research is needed to further elaborate personal experiences with healthcare and examine how health system factors reinforce and/or mitigate positive health-seeking behaviours, including timely use of health services.

## Data availability statement

The data analyzed in this study is subject to the following licenses/restrictions: the datasets used and/or analysed during the current study are not publicly available as the authors are still using the data for other analyses. However, data will be made available from the corresponding author upon reasonable request. Requests to access these datasets should be directed to DH, hotchkis@tulane.edu.

## Ethics statement

The studies involving human participants were reviewed and approved by the Ministry of Health, Jamaica and the University of the West Indies/University Hospital of the West Indies Ethics Research Committee (ECP 169, 14/15). Written informed consent to participate in the study was obtained from all adult study participants and, for participants under 18 years of age, was provided by the participants’ parent and/or legal guardian.

## Author contributions

LC conceptualized the paper, developed the analytic strategy, and analysed the data and interpreted the findings. CC-M, KT, JG, JH, and DH critically revised the paper for important contextual background and intellectual content. All authors contributed to the article and approved the submitted version.

## Funding

This secondary analysis research received no specific grant from any funding agency in the public, commercial or not-for-profit.

## Conflict of interest

The authors declare that the research was conducted in the absence of any commercial or financial relationships that could be construed as a potential conflict of interest.

## Publisher’s note

All claims expressed in this article are solely those of the authors and do not necessarily represent those of their affiliated organizations, or those of the publisher, the editors and the reviewers. Any product that may be evaluated in this article, or claim that may be made by its manufacturer, is not guaranteed or endorsed by the publisher.

## Glossary

AIC: Akaike Information Criteria
BIC: Bayesian Information Criteria
CD: Compact disk
CI: Confidence interval
COPD: Chronic obstructive pulmonary disease
HRQoL: Health-related quality of life
IPAQ: International Physical Activity Questionnaire
JADEP: Jamaica Drug for the Elderly Programme
JHLS-II: Jamaica Health and Lifestyle Survey 2007/2008
LCA: Latent class analysis
MCS-12: Mental Component Summary
MET: Metabolic equivalent
NCD: Non-communicable disease
NHF: National Health Fund
OR: Odds ratio
PCA: Principal components analysis
PCS-12: Physical Component Summary
